# Stigma-Generating Mechanisms in Families Enrolled in a Pediatric Weight Management Program: A Qualitative Study of Health Identities and Healthcare Authenticity

**DOI:** 10.3390/children11010046

**Published:** 2023-12-29

**Authors:** Mie Madsen, Lene Michaelsen, Patricia DeCosta, Dan Grabowski

**Affiliations:** 1 Department of Prevention, Health Promotion and Community Care, Copenhagen University Hospital—Steno Diabetes Center Copenhagen, Borgmester Ib Juuls Vej 83, 2730 Herlev, Denmark; patricia.enebaer.irene.decosta@regionh.dk (P.D.); dan.grabowski@regionh.dk (D.G.); 2 The Centre for Children and Youths Health, Mimersgade 47A, 2nd Floor, 2200 Copenhagen, Denmark; lene.michaelsen@kk.dk

**Keywords:** childhood obesity, weight stigma, family-focused interventions, pediatric health care, prevention, qualitative research, familial health identities, authenticity

## Abstract

In recent years, there has been increased awareness of obesity as a condition that carries a high level of stigma, as well as growing recognition of its prevalence and harm. Despite the increasing body of research on this topic, there is a gap in the literature regarding mechanisms that generate or exacerbate perceptions of weight stigma, especially within families and pediatric healthcare settings. The present study aims to identify potential stigma-generating mechanisms by focusing on inter-relational dynamics within these contexts. We conducted in-depth, semi-structured interviews with 11 families and analyzed the data by applying sociological theories on health identities and authenticity. Our study found four themes that represent potential stigma-generating mechanisms by being explicitly related to familial health identities and healthcare authenticity: (1) negotiating and reconstruction familial self-understanding, (2) between guilt, shame and conflicts, (3) navigating weight perceptions, and (4) the necessity of positivity and relevance. Our study shows the complexities of weight stigma within family and pediatric healthcare settings, emphasizing the need for sensitive and tailored support, as well as the value of working authentically as crucial aspects in preventing and/or reducing stigma.

## 1. Introduction

Childhood obesity among children and adolescents is a substantial health concern worldwide and has been linked to an increased risk of developing comorbidities [1]. Research has shown that, during childhood and adolescence, obesity can contribute to the onset of conditions such as type 2 diabetes and hypertension and have a significant impact on overall quality of life [2,3,4]. Furthermore, studies have demonstrated that the long-term health consequences of obesity, both physical and mental, can persist into adulthood [5,6].

In recent years, growing attention has been paid to the subject of obesity as a condition that carries a high level of stigma, along with an increased recognition of the prevalence and harm of weight stigma [7,8,9]. Studies have therefore highlighted weight stigma as a psychosocial factor to be considered in the treatment of obesity [10,11,12,13].

Weight stigma, weight bias, or weight-based discrimination can be understood as negative weight-related attitudes and beliefs that are socially constructed and manifested through stereotypes, marginalization, and social deviance [14,15]. Weight stigma is typically directed at individuals with overweight and obesity because they deviate from society’s constructed understandings and beliefs regarding what an ideal body weight and shape should be. This phenomenon is particularly prevalent in the Western societies’ health paradigms [16,17].

Link and Phelan [18] conceptualized stigma as a social process involving four primary components that occur among individuals and are influenced by power dynamics. According to their framework, stigma encompasses (1) labeling: people identify and categorize differences among individuals, (2) stereotyping: dominant cultural beliefs associate the labeled individuals with undesirable traits, (3) separation: labeled individuals are segregated into distinct groups, creating an “us versus them” dynamic, and (4) status loss and discrimination: labeled individuals face discrimination and experience a decline in social status, resulting in unequal positions. According to Link and Phelan [18], stigma exists along a continuum, and not all four components need to be equally present in the process of stigmatization.

Link and Phelan [18] also emphasized the socio-psychological aspects of stigma, particularly the concept of embedded self-stigma. This phenomenon pertains to individuals who, when exposed to weight stigma in their environment, may internalize these weight-related beliefs and attitudes, also referred to as internalized weight stigma [14]. As a result, individuals may incorporate these unfavorable perceptions and stereotypes into their self-perception, ultimately leading to self-devaluation [18].

A growing body of research has examined weight stigma as a distinct factor contributing to adverse psychological and mental health consequences and behaviors [9,14,19,20,21,22]. Studies have demonstrated that weight stigma within educational settings can lead to reduced academic performance and limited social involvement [4,23]. Similarly, it has been documented that weight stigma is a central factor causing children and adolescents to isolate themselves from friends and family, experience loneliness, depression, and anxiety, endure low self-esteem, contend with body dissatisfaction, and foster negative body image perceptions [22,24,25,26]. Furthermore, research has shown that weight stigma is a factor that can promote unhealthy behaviors. Children and adolescents with obesity who experience weight stigma are at a high risk of developing eating disorders, often avoid participating in physical activities, and have limited engagement in preventive and therapeutic healthcare services [27,28]. This body of evidence strongly indicates that weight stigma not only hinders efforts to improve health and well-being but also increases the risk of behaviors that perpetuate and exacerbate obesity.

The vulnerability of children and adolescents to adverse social interactions is heightened during their developmental stage, which is characterized by substantial psychological, physical, and social changes [29,30]. Unfortunately, weight stigma is pervasive and can be encountered in various relationships and contexts, including schools and leisure activities [31,32,33], healthcare systems [34], homes [35], and the media [36,37]. Its manifestations are diverse, encompassing explicit behaviors like teasing, bullying, and harassment, as well as subtler, implicit expressions related to marginalization processes, such as avoidance, disregard, and differential treatment [26].

As regards addressing weight stigma, the family plays a crucial yet complex role. Family dynamics significantly influence a child’s identity and self-concept [20,38], with parents being instrumental in creating supportive home environments and shaping children’s health behaviors [39,40]. This has led to a great interest in involving parents and families in programs aimed at preventing or addressing childhood obesity [41,42,43,44]. However, research has also identified parents and other family members as potential sources of interpersonal weight bias [20,45,46,47]. 

Pediatric healthcare settings have also emerged as arenas where weight stigma may be encountered by children and their families [16,48,49]. However, research on stigmatization in healthcare settings has primarily focused on adults with obesity, leaving a gap in our understanding of the pediatric healthcare context [50]. Nevertheless, pediatricians, as key advocates for children’s health, are tasked with the delicate responsibility of addressing weight-related issues while simultaneously promoting resilience and providing support to children and their families in the face of stigma [51].

Despite the increasing volume of research on weight stigma, our knowledge about its implications in the context of programs and pediatric health care remains limited [50]. Puhl [52] emphasized that weight stigma creates barriers to providing effective health care, such as fostering poor communication between patients and professionals, reducing the quality of care, and prompting healthcare avoidance. A recent study by Hoeeg et al. [53] explored the impact of stigma on interventions aimed at preventing and treating childhood overweight and obesity through a family-based interventions. It showed that mechanisms of stigmatization hinder the effectiveness of these interventions. Additionally, studies by Hoeeg et al. [54], Roberts et al. [55], and Roberts et al. [56] have shed light on how blame, parental conflicts, and disagreements regarding a child’s weight and weight management approaches can escalate tension and stress within families. These conflicts can exacerbate the child’s distress and feelings of helplessness, consequently contributing to the development or intensification of the perception of weight stigma. 

There is a lack of comprehensive research on the underlying mechanisms that generate or amplify the perception of weight stigma within family and pediatric healthcare settings, as well as the ways in which these aspects are interconnected. Identifying and understanding these mechanisms are crucial to reducing and preventing weight stigma [57].

In the present paper, our main objective is to study stigma within the context of family programs and pediatric healthcare settings by focusing on inter-relational dynamics that may determine the development of stigma and/or amplify the perception of it. We will do so by studying families’ experiences of their participation in a pediatric family-focused weight management program. 

The paper will improve our understanding of the underlying mechanisms that may potentially generate stigma or contribute to its development. It provides valuable insights for developing more effective and sensitive programs, especially for healthcare professionals working with children and families facing the challenges of obesity. In the following section, we will outline the theoretical foundation for our analysis. 

## 2. Theory

In our analysis, we will focus on two interconnected theoretical elements: familial health identity and healthcare authenticity. 

The concept of health identity draws on Waterman’s theories on delineated self-definitions [58], Taylor’s social imaginaries [59,60], and Luhmann’s expectational structures [61]—with the definition*: “People’s observations and expectations concerning their own health, their knowledge about health and in what ways their health is related and comparable to the health of others”* [62]. The definition emphasizes the development of individual health identities through personal observations (and consequent meaning-making) of communication. Health identity is expressed through expectational structures and social imaginaries, and it influences health values, health beliefs, and health choices. Health identity serves as a way of orienting and navigating in the complexities of health communication, health information, and potential health behaviors [62,63].

The concept of familial health identity enables us to examine the complex inter-relational dynamics that may determine the development of stigma and/or amplify the perception of it in the context of a pediatric family-focused weight management program. Concretely, it allows us to explore how families (and individual family members) perceive and construct their own health identities in the context of obesity treatment. It recognizes that families develop shared beliefs, values, and behaviors related to health and weight management. By understanding how these identities are formed and how they may be influenced by societal norms and expectations, we gain insight into the potential mechanism of weight stigma among families.

The concept of authenticity, as described by Barab et al. [64], is a process that is realized through engagement in meaningful tasks and practices. From this perspective, individuals can experience authenticity when they are able to integrate health information or potential health behaviors into their everyday lives in a meaningful manner [64]. Petraglia [65] conceptualized authenticity as a prospective means of transforming knowledge into communicative action, emphasizing *“that the legitimisation of information as authentic is not a matter of possessing factual or technically correct information but rests on our belief that the information conforms with our sense of who we are and what we know”* [65]. Noddings [66] defined authenticity by distinguishing between rule-bound caring and empathic caring, and Cranton and Carusetta [67] argued that authentic educators must demonstrate communicative consistency by aligning their values with their actions. Additionally, Kreber et al. [68] described authenticity as a multifaceted phenomenon, emphasizing it as a process of negotiating meaning. Guttman et al. [69] argued that the authenticity associated with caring creates a sincerity that strengthens the information and communication, which is then perceived as credible. 

These diverse definitions are closely linked to facets of communication and form the background against which we have framed a health education model that was initially derived from empirical studies conducted on health education courses [70] and here adapted to the context of a pediatric family-focused weight management program. The four categories are (1) authentic relationship between family and healthcare professionals: creating a sense of genuine caring, as opposed to rule-bound caring, (2) authentic healthcare professional: owing to the position he/she represents or because he/she possesses important skills or knowledge, (3) authentic thematic contents: creating a sense of thematic applicability to and usability in daily family life, and (4) authentic activities: creating a sense that the activities are meaningful [70] (pp. 94–97). These four authenticity categories form the theoretical framework underpinning the analysis.

Combining the theories on familial health identity and healthcare authenticity provides us an informed analytical framework with which to capture potential stigma-generating mechanisms among families participating in a family-focused obesity treatment for children by highlighting complex inter-relational dynamics that influence the development of potential stigma and/or amplify the perception of stigma.

The concept of familial health identity allows us to explore how families perceive and construct their own health identities within any given context. It recognizes that families develop shared beliefs, values, and behaviors related to health and weight management. By understanding how these identities are formed and how they may be influenced by societal norms and expectations, we gain insight into the potential mechanism of weight stigma within the family. The concept of healthcare authenticity enables us to examine the interactions between health care and families, recognizing that experiences with health care can have a significant impact on individuals’ and families’ perceptions and attitudes toward their health conditions. 

In summary, the two theoretical concepts allow us to capture the complex interplay of dynamics that shape the development of stigma and potentially amplify its perception in a concrete setting. 

## 3. Setting and Methods

We chose to apply a qualitative and theoretical approach with a distinct focus on families’ experiences, perceptions, and thoughts, enabling us to understand how families experienced their participation in a specific pediatric weight management program with an additional focus on how this resonated with their daily life.

### 3.1. The Program

The program under study is a family-focused approach to the treatment of childhood obesity for children aged 2–16 in a large urban municipality in Denmark. Approximately 500 families are enrolled in the program every year. The children often live with additional psychosocial challenges, and the parents often live with obesity themselves. Most of the families are characterized by low levels of education; approximately 50% of the parents are unemployed, and approximately 60% of the families belong to ethnic minorities.

The goal of the program is to promote healthy weight development that can support a childhood characterized by physical, mental, and social well-being. The treatment is based on the understanding that healthy weight development is influenced by a multitude of factors in the children’s lives. In the program, there is an explicit focus on seven factors: (1) health, (2) diet, (3) physical activity, (4) family, (5) mental health, (6) relations, and (7) body. The emphasis of each factor varies from family to family, but experiences from the program show that all factors are, in fact, important for all the families with regard to creating a solid foundation for healthy weight. The treatment is carried out by an interdisciplinary team of doctors, nurses, dieticians, physical educators, and psychologists.

### 3.2. Recruitment, Participants, and Interviews

The interviewed families were purposefully sampled by the healthcare professionals working in the program. The only criteria for inclusion were that the child and at least one of the parents spoke Danish well enough to be interviewed and that the child was old enough to take part in the interview. Based on previous studies and our experience with similar interviews, we decided that the cut-off would be 9 years of age. In families with children younger than 9, the dynamics (especially regarding autonomy and emancipation) are significantly different from families with older children [53]. Furthermore, we wanted an equal distribution of boys and girls represented in the study.

In practice, the healthcare professional would ask the families if they were interested in being interviewed. If they were, the healthcare professional would pass on the families’ contact details to one of the researchers, who would then contact the families with detailed information.

After the first 11 interviews, the research team assessed the level of data saturation [71]. It was agreed that no new overarching themes had emerged during the last three interviews. The team also assessed the heterogeneity of the recruited families and agreed that the families represented the overall composition of enrolled families to a satisfactory degree. Recruitment was therefore stopped. Table 1 represents an overview of the participants.

The interviews were semi-structured around the following overarching themes: (1) the consultations, (2) weight management tasks at home, (3) everyday life, (4) familial health, and (5) self-understandings. 

The interviews were audio recorded, and the average duration was 59 min. All interviews were carried out in the participants’ homes.

We consulted the COREQ established standard for reporting qualitative research throughout the research process. We live up to all items on the checklist, with the exception of returning the transcripts to the participants for comments. We chose not to do that because it can be a cause of embarrassment and anxiety for interviewees who are vulnerable and prone to self-stigmatization [72]. 

### 3.3. Ethics

Written informed consent was obtained from all parents and children prior to the interviews. All parents and children were informed that their involvement in the study was voluntary and that they could withdraw at any time without consequences for their child’s treatment. To ensure participant confidentiality and protect anonymity, real names were removed in reporting and identifying details were omitted. The study adhered to the ethical guidelines laid down in the Declaration of Helsinki and was approved by the Danish Regional Data Protection Agency (No: P-2021-678) [73].

### 3.4. Analysis

The interviews were transcribed verbatim and analyzed using radical hermeneutics. This analytical approach comprises a set of principles guiding content analysis, with a focus on maintaining a perpetual balance between theory, methods, and data. It recognizes that all of these elements influence each other in an interconnected process. Employing radical hermeneutics also involves a continuous alternation between analyzing and interpreting, necessitating the presentation of interpretive aspects alongside the results.

The methodology consists of three steps of data analysis. The first step involves reading the data with a view to observing specifically selected differences in them. This observation per se constitutes an interpretation rather than a description, aiming to simplify the complexity of the data. Elements falling within the scope of distinctions selected by the interpreter are then extracted from the data. The second step involves making these elements the subject of interpretation as an observation of the differences employed. The third step involves interpreting the sum of these differences [74]. 

In the current analysis, the initial step concentrated on extracting matters relevant to stigma. This coding was undertaken manually. The second step involved analyzing and interpreting the extracted data using the theoretical background presented above, and this step revealed the four main themes presented in the results section. The final step was then a separate interpretation of the data within each category—materializing as the findings presented for each of the four themes. 

## 4. Findings

Our analysis enabled us to identify four overarching themes that are all connected to our research focus on stigma-generating mechanisms: (1) negotiating and reconstructing familial self-understandings, (2) between guilt, shame, and conflicts, (3) navigating weight perceptions, and (4) the necessity of positivity and relevance. The four themes explore the dynamics and mechanisms that highlight potential stigma-generating mechanisms. The occurrence of explicit stigma such as bullying or social exclusion is not the main focus of our study, but rather we focus on the underlying and implicit dynamics and mechanisms that can contribute to perceived stigma.

### 4.1. Negotiating and Reconstructing Familial Self-Understandings

Participating in a family-focused weight management program involves a diverse journey for the families, one that is directed by and impacts their familial self-understanding. Throughout the program, families often experience a strengthened family bond by adopting a collective approach, which enhances their understanding of their shared familial identity. However, the families are also challenged in their existing understandings and expectations concerning their own health behavior, causing them to negotiate and reconstruct their familial health identities through the process of engagement in the program. When interviewing the families, it became evident that the program represents a shared endeavor within the families to achieve positive outcomes. One mother exemplifies:

“*It’s not just you* [the son] *who takes part in the program, it’s the whole family. It’s good for the entire family. That became really evident during COVID, where we went out for walks a lot, and we did all those things together, and Ida* [sister]* was also involved. Yes, it became more of a family thing*.”

(Family A, mother)

This mother emphasizes how participating in the program nurtures a sense of togetherness through shared experiences. Although the program is tailored to address particularly the child’s obesity, the mother’s statement underscores the significance of involving the entire family, recognizing the family as a collective entity for the program. The child’s participation in the prograu becomes a collective project for the family, fostering a shared understanding of health and cultivating collective familial health behavior. Several families recognize the benefits of perceiving the family as a collective entity for the program. One boy expresses how his participation in the program became a collaborative familial project, creating a profound sense of support:

“*This is what helps the most. For example, when I only eat a little bit, then everyone eats a little bit… they don’t eat a lot, and I get a little. If I take a little, then everyone takes a little, and then it’s okay*.”

(Family E, 12-year-old boy)

The boy’s quote underscores the formation of a novel familial social norm, wherein the consumption of smaller portions is deemed acceptable provided that all family members participate equally. This experience serves as a positive reinforcement for the boy’s familial self-understanding, highlighting the role of shared responsibility and collective effort in establishing a new advantageous eating practice within this context. Engagement in equal and collective participation acts as a mechanism that drives the development of a new familial health behavior. 

However, participation in the program also presents challenges to some families’ self-understanding. Particularly, the realization of participating in the program is something that affects the families’ self-understanding, initiating negotiation of familial health identity formation. One mother says:

“*I mean, it’s worth trying *[participating in the program]* once you’ve accepted the thought: Are we not doing well enough on our own?*”

(Family A, mother)

Several of the parents experience being impacted by a sense of inadequacy while participating in the program—by the feeling that they have not done well enough. When participating in the program, several of the parents deal with a sense of self-reflection and self-doubt. This signifies a level of self-criticism and apprehension regarding their own capabilities, which, in turn, challenges their pre-existing familial self-understanding. For many families, this feeling of inadequacy and self-criticism can act as a barrier to their involvement in the program. For most families, the realization that they need help is something they must process, and afterward, they manage to look positively ahead. The mother adds:

“*A lot of what we’ve done now is something we were already doing before, but it’s just different to be part of a program, where someone can see things from a different perspective or come up with ideas about something you haven’t thought of yourself*.”

(Family A, mother)

The mother highlights the importance of engaging in the program. While some of the actions they took were already part of their routine, being involved in the program offers a different dynamic. It allows for new insights, diverse perspectives, and the possibility of discovering new ideas that may not have been considered before. Openness to these new perspectives allows them to be integrated into the families’ self-understandings and can contribute to a reconstruction of the families’ health identities. However, there is considerable variation in how the families interpret and respond to the realization and acknowledgment of needing assistance in addressing their child’s obesity, which manifests either in a positive or negative manner. Moreover, the parents employ different strategies when confronting potential challenges related to their established familial self-understanding and health identities. A quote from one mother illustrates this dynamic:

“(…) *but it’s just difficult, and I think you also see and sense this here in our house. You don’t automatically think that this is just a family, constantly eating marshmallows and peanuts, pouring all sorts of things into their mouths. We actually live quite healthy. I would have liked to be a chef, and I’ve just published my second cookbook. So, I’m completely immersed in food, and if you look around, there are cookbooks everywhere, and I think it’s a genuine joy when people gather and eat a great meal together. And also, we grow cabbage and always strive to have a healthy foundation*.”

(Family B, mother)

Upon entering the program, families bring with them their distinctive behavior, norms, and understandings related to health, influencing their familial self-understanding and familial health identities. The mother’s viewpoint reflects a particular understanding of ‘healthy’ versus ‘unhealthy’ behaviors, encompassing notions of legitimate and illegitimate health practices. She categorizes eating marshmallows, peanuts, and overeating as ‘unhealthy practices’. This classification runs contrary to the mother’s perception of her family’s health identity, characterized by their dedication to cultivating a health-conscious lifestyle. 

Moreover, the mother’s conscious emphasis on their family’s robust commitment to healthy eating can also be interpreted as a way for her to maintain her own self-understanding. This emphasis may serve as a subtle strategy to deflect external judgments or stereotypes related to their health behaviors, thereby underscoring her resolve to uphold her family’s health-conscious identity.

Notably, the fear of judgment and being stereotyped is a prevalent concern among families, and many parents carry feelings of guilt and shame with them.

### 4.2. Between Guilt, Shame, and Conflicts

In general, the families express their satisfaction and happiness with participating in the program. However, there are also aspects that are experienced less positively. When their families’ self-understanding is challenged and significant behavioral changes may be required, conflicts can arise. These conflicts can manifest in various ways among the families, but a recurring conflict area is when it explicitly concerns the child:

“*But it’s also about the fact that we end up in conflicts with our child… that it turns into a situation where he, as he himself says, feels forced by us—it’s really difficult. We as parents are compelled from their side, and then I force a certain behavior on him, and then it escalates into a conflict*.”

(Family H, mother)

To some extent, this mother holds the healthcare professionals responsible for this conflict. By implicitly placing blame on the professionals, the mother may find it more manageable to understand and accept the circumstances, and this becomes her way of navigating these difficult situations. However, the mother’s feeling of being “forced” may also serve as a mechanism that challenges the authenticity of the relationship between the family and the healthcare professionals.

During the interviews, we also see how families, in particular mothers, navigate emotional challenges related to feelings of guilt and shame, which deeply affect them. The interviews reveal several examples where parents label themselves as bad, unhealthy, or inadequate at maintaining their families’ health. One example is from this mother’s statement:

“*I take it more personally because I feel guilty about it. I prepare the food at home, and it’s me who does the grocery shopping, and I keep thinking about what I might be doing wrong. So, I feel a great responsibility for it. And every time he’s measured or when he’s gained weight, I take it very hard. It really, really [cries] upsets me, and I can’t stop thinking about it because I’m genuinely concerned about his health and weight*.”

(Family H, mother)

In this quote, we see how the mother assumes sole and personal responsibility for her child’s health and weight. She expresses a strong sense of guilt, feeling personally accountable for the food choices the family makes. The feeling of guilt suggests a self-imposed burden, possibly driven by societal expectations and standards concerning parenting and health. Furthermore, the mother’s guilt indicates an internalized perception of personal failure or inadequacy in fulfilling her role as a parent in maintaining the child’s well-being. This example of emotional response and preoccupation with the issue highlights the serious guilt and blame related to the complex relationship between parenting and childhood obesity. The experience of guilt and shame is also something that subtly permeates the consultations between the families and the involved professionals. One mother exemplifies this:

“*I can see in Leo that he doesn’t feel like telling them that we might have a package of cookies at home. They’re overly concerned about what we have in our home. Of course, it’s because they want to help, and that’s fine, but I believe it’s too intrusive to ask, ‘Are there any carrots in the refrigerator?’ It’s a matter of privacy. It’s just a bit too much, and I can also see that Leo is tired of it—of having to tell them every time what we have in the fridge*.”

(Family H, mother)

The discussions about food and eating habits are subjects that can evoke feelings of guilt and shame, which in turn contribute to challenging dynamics between the families and the professionals. The mother’s frustration with the professionals’ emphasis on the contents of their home, especially regarding unhealthy food items like cookies and candy, can be interpreted as a means of avoiding potential judgment on the part of the professionals and preventing guilt. The feeling of a constant need to explain or justify the family’s food choices may contribute to a sense of shame or discomfort. The mother’s frustration may indicate a perception of being judged by the professionals. 

It is not only the parents who express feelings of guilt and shame regarding their participation in the program:

“*I only keep it to myself* [participating in the program]*, my mother, my stepmother, and my father. They are the only ones I’ve mentioned it to. I don’t feel like telling my friends because I think they might gossip to others*.”

(Family K, 9-year-old girl)

The girl does not explicitly mention guilt, but her reluctance to share the family’s participation in the program implies a sense of implicit guilt or embarrassment. Her decision suggests a desire to avoid potential shame. It is evident that the girl’s apprehension is influenced by prevailing social norms and expectations, wherein it is not socially accepted to engage in a weight management program. For the girl, weight management seems to be a sensitive and delicate topic, as well as a potential criterion for exclusion due to societal health expectations and understandings.

### 4.3. Navigating Weight Perceptions 

During the interviews, no explicit questions were asked regarding the families’ encounters with discussing weight or being weighed. However, the issue of weight and the related discussions were subjects that resonated with all families. It became evident that a focus on weight and the act of being weighed are complex issues. One girl’s statement shows some of the intricacies involved:

“*Well… I prefer it when we talk about all the good things. Yes, it’s about me being overweight, but that they don’t mention it directly, and that they still, you know—so it doesn’t become like ‘you’re overweight!’, but that they talk about health—what we can do better, you know*.”

(Family K, 11-year-old girl)

Being labeled as “overweight” is challenging for the girl, reflecting that the concept of being overweight is not a neutral and ‘innocent’ experience. It is evident that weight-centrism and being labeled as overweight have negative connotations and are closely intertwined with the girl’s self-perception, potentially contributing to feelings of discomfort. Additionally, the girl’s statement reflects how weight-centrism fosters a process of identity negotiation. The girl finds herself navigating between acknowledging that she is overweight and wanting to avoid being labeled and treated as “overweight”. This shows that the girl is aware that being overweight is an undesirable trait, which contributes to her fear of being labeled as overweight. As a result, it prompts the girl to emphasize “all the good things”, which, from her perspective, involves avoiding a focus on weight. Building on this, the girl experiences a positive impact on her engagement in the program, with professionals addressing her weight by adopting a holistic approach centered around health and overall improvement rather than solely concentrating on weight. By doing so, the girl experiences that the program implicitly addresses the issue of her being overweight, but without labeling her as overweight. A mother also shares how she experiences the shift toward discussing health rather than weight as very positive:

“*That’s actually one of the things I really appreciate about it in there, that it’s not so much about ‘what do you weigh?’ but it’s more about ‘how healthy are you?’ and ‘can we do something to make you healthier?’ and that health is something that you can’t see on the outside. It’s what you can see on the inside, and I think that’s really great. We focused a lot on weight at home. That was something we could measure ourselves, but that’s not what it’s all about*.”

(Family A, mother)

This shift toward discussing health rather than weight is an approach that appears to lift the families up and motivate their participation in the program. The mother also reflects on their previous experiences at home, noting that weight was often a significant focus. However, she expresses a shift in perspective. This indicates that the families’ participating in the program contribute to reconstructing their health identities, with an emphasis on prioritizing health instead of ‘weight’. Weight and the act of being weighed, however, present a paradox. Although families express their positivity toward adopting a holistic approach to health and not solely concentrating on weight, weight and weight loss continue to receive significant attention, occupying a prominent place in the minds of the participating families. One mother highlights this:

“*It’s difficult because perhaps there shouldn’t be so much focus on weight. Because, at least Line* [daughter]*, she thinks about it too much and talks about it a lot, even though I’ve tried to say all the time that it’s not about how much you weigh, because you can weigh a lot and still be healthy, and it’s about being healthy, but that doesn’t stick in her memory—and it’s also difficult not to talk about weight because that’s what you assume*…”

(Family C, mother)

The mother addresses the difficulty of avoiding discussions about weight, as it is commonly assumed to be a significant aspect of health. She expresses uncertainty about how to approach the topic differently, which highlights the complexities involved. Particularly intriguing in the mother’s statement is also her statement concerning how weight-centrism specifically affects her child. Despite efforts to maintain a focus on health, weight remains an overshadowing indicator. It goes beyond being a mere numerical value and becomes a pervasive form of objectification, especially affecting the girl and her self-perception. 

### 4.4. The Necessity of Positivity and Relevance

As emphasized in the preceding themes, weight emerges as a sensitive topic for the participating families, encompassing a multitude of complexities and challenges. The professionals must navigate and address these intricacies and difficulties, and this is where healthcare authenticity becomes particularly relevant as a concrete tool to manage these emotional challenges in a positive and supportive way. The experience of one boy highlights the positive impact of healthcare authenticity on his engagement in the program:

“*Well, in the beginning, I was negative about it. It was like, ‘uh… now we have to go again’ (…) but I actually think it’s gone pretty well. And it’s quite fun to be there, it’s kind of… nice*.”

(Family A, 12-year-old boy)

The boy experiences a shift in his perspective as he becomes more involved in the program, signifying a change in attitude. This transformation shows how the process of engagement allows the boy to reassess and modify his existing perceptions. It becomes evident that the boy finds meaning in participating in the program. The opportunity for enjoyable experiences positively influences him, indicating that the boy experiences authentic activities. Additionally, one mother expresses her positive attitude toward participating in the program and acknowledges the professionals’ ability to navigate and support her feelings regarding her child’s obesity:

“(…) *and then suddenly, time has just slipped away from us because there are so many things we want to talk about (…) You can’t sense any hurry from their side. They don’t give the impression that we need to hurry because someone else is coming. You feel like you have plenty of time to resolve things, to discuss them thoroughly. I remember a time where I was very frustrated and had a lot of guilt as a mother, thinking it was all my fault. Then they offered me individual conversations where we discussed things—and I found that to be really, really comforting*.”

(Family E, mother)

This mother finds her participation and the discussions meaningful. The absence of a rushed atmosphere and the perception of having sufficient time to address concerns help to create a comfortable environment for open dialogue. A recurring pattern among the families is also the sense of security, as they feel comfortable and at ease when participating in the program and engaging in conversations with the professionals:

“*Well, I’m kinda’ happy when we go there because I feel safe when we’re there, and they’re really, really nice to me. They talk respectfully, and they do tell us the things we need to do, but most of the time, they also say things that are good. So, every time they say something, it’s something good—and something we can do better*.”

(Family K, 11-year-old girl)

The girl’s quote exemplifies the advantages of creating a safe environment and fostering a trusting relationship between families and professionals. When families feel secure, this cultivates an atmosphere where they are more open to receiving advice and constructive criticism. Likewise, it demonstrates the potential to address sensitive subjects like guilt and shame in a positive and supportive way.

There are also other ways in which authenticity becomes apparent in the professionals’ work with the participating families:

“*I think what you* [the child] *appreciate is that she* [the professional] *herself has children, so she knows what it’s like to be 12 years old. It’s easy for you to relate when she talks about her daughter, and she knows* that *you’re probably sitting on the couch with a screen in front of you. She has great insight into the child’s world and what can be difficult and challenging when you snack too much during the afternoon. She can sense what challenges you’re facing*.”

(Family A, mother)

The professional’s first-hand experience of being a parent allows her to relate to the child’s perspective and to establish an identifiable presence within a relevant context, thereby enhancing the credibility and legitimacy of the role as a professional. Moreover, this fosters reciprocity between the family and the professional, ultimately enhancing the relevance of the professional’s guidance and making it more valuable for the parents and the child.

## 5. Discussion

In the present study, we have identified and explored potential stigma-generating mechanisms and dynamics. We have approached this through a theoretical lens of familial health identity and healthcare authenticity. Our focus has not been on the overt occurrences of explicit stigma but, more significantly, on the underlying and implicit dynamics that can contribute to the perception of stigma. 

Through interviews with families, we have gained valuable insights into the daily lives of families dealing with obesity and their participation in a pediatric family-focused weight management program, as well as their family narratives. This understanding has allowed us to identify four themes: (1) negotiating and reconstructing familial self-understandings, (2) between guilt, shame, and conflicts, (3) navigating weight perceptions, and (4) the necessity of positivity and relevance. 

The four themes represent dynamics that encompass a dual trajectory concerning possible mechanisms that have the potential to either reduce or exacerbate the perception of stigma within the relationship between families and healthcare professionals if not addressed promptly.

### 5.1. Negotiating and Reconstructing Familial Self-Understandings

Throughout the analysis, we found variation in how families interpret and respond to the recognition of their need for assistance in addressing their child’s obesity. This variation manifests itself in both positive and negative ways, reflecting the complexity of familial responses to health programs in pediatric healthcare settings.

Several families recognized the benefits of approaching the program as a cohesive family unit. The transformative potential of collective family participation became evident, not only in cultivating healthier behaviors but also in mitigating the potential for perceived stigma associated with those practices. Through shared responsibilities and cooperation, families can potentially navigate the challenges of managing weight and health together more effectively. Furthermore, this collective experience can be viewed as a mechanism that serves to reduce stigma. This insight invites healthcare professionals to recognize and leverage the positive influence of these familial dynamics in their interactions with families.

In contrast, many parents found it challenging to acknowledge that they need help with managing their health and addressing their child’s obesity. This acknowledgment often contradicts the narrative they have constructed, leading to internal conflicts when attempting to integrate the weight management program’s principles, approaches, and values into their familial health identity. Consequently, this struggle can foster a subtle distance in the family’s involvement in the program, creating narrative confusion and the potential for stigma-related barriers. These barriers may prevent parents from fully embracing the program and its principles. These findings align closely with the research of Hoeeg et al. [53], highlighting the role of stigmatization as a crucial factor influencing efforts to address childhood overweight and obesity through family interventions, as well as with Puhl [52], who emphasizes that weight stigma poses obstacles to providing effective care. Building upon this, there is a need for heightened awareness regarding the intricate interplay within families and their interactions with pediatric healthcare practices. 

### 5.2. Between Guilt, Shame, and Conflicts

A recurring theme emerging from our analysis was the presence of conflicts within families, notably in situations involving the children. These conflicts often revolved around the child’s perceived feeling of being “forced” to make behavioral changes. This created tensions within the family. Some parents attributed some of the pressure to healthcare professionals, implying that their guidance contributed to the escalation of conflicts. It is important to note that this attribution of blame to healthcare professionals might serve as a coping mechanism. In navigating difficult family situations fraught with conflicts, especially derived from underlying feelings of guilt and shame, it may be more manageable to place some responsibility on external factors, in this case, the professionals, rather than solely internalizing the challenges.

We found that many parents experience a significant amount of guilt concerning their family’s overall health challenges. This burden is particularly evident among mothers, who are often responsible for grocery shopping and cooking. These findings align with a study conducted by Roberts et al. [56], which highlights how parents express guilt regarding their adolescent’s weight, with mothers in particular feeling the burden of daily weight management. In our analysis, the burden of guilt furthermore manifests as shame and becomes something that is difficult to talk about. Our analysis shows that, in families where ‘unhealthy practices’ and obesity are associated with personal guilt and shame, there are clear tendencies for this to be internalized as intra-familial stigma, which is consistent with the findings of Hoeeg et al. [54], who identified that stigmatization emerged when parents placed blame on the child with obesity and attributed responsibility for their own health and weight loss to them. It is apparent that this internalized stigma affects the family’s ability to develop and sustain coping mechanisms that can protect them from external categorization and stigmatization. 

### 5.3. Navigating Weight Perceptions

While the majority of families recognize the significance of being weighed in their efforts to address obesity, the topic of weight is a sensitive and delicate one for the participating families. Weight, in this context, is not a neutral or innocuous concept; instead, it serves as a means of categorization, with the numerical value of weight acquiring a social connotation. This ‘label’ has the potential to classify individuals as either socially accepted or socially marginalized, influenced by prevailing societal and cultural norms and perceptions related to health and weight [75]. The act of being weighed thus functions as a mechanism that contributes to and/or amplifies the sense of perceived stigma through the feelings of guilt and shame. Consistent with this, Roberts et al. [56] found that addressing weight inappropriately often led to feelings of shame, ineptness, and blame in both the child and parents. Moreover, numerous studies have similarly underscored the influence of body weight on individuals’ social identities, emphasizing how weight stigma represents a threat to one’s social identity [10,76,77]. This implies that individuals have concerns about experiencing devaluation, discrimination, rejection, or negative stereotypes [78]. In our study, we observed how, for an 11-year-old girl, this manifested as a process of identity negotiation. She found herself navigating between acknowledging her condition as overweight and desiring to avoid being labeled as ‘overweight’ in order to ‘fit in’ with society’s constructed understandings and beliefs regarding the ideal body weight and shape. Furthermore, we also observed, for another 9-year-old girl, how these concerns about experiencing devaluation, discrimination, rejection, or negative stereotypes manifested as a reluctance to share her and her family’s participation in the program.

Several families reported a transformative shift in their approach to weight and being weighed during their participation in the program. These families reframed the act of weighing and the associated focus on weight in a more constructive and positive way by embracing a health-focused, weight-inclusive mindset. Numerous studies have stressed the significance of shifting from a sole emphasis on weight to an approach to health care centered on holistic health that incorporates considerations related to weight. This shift has been recognized as a strategy to mitigate weight stigma within clinical settings, ultimately leading to improved healthcare experiences for individuals with overweight and obesity [79,80,81]. However, it is important to note that the research in this area lacks a clear consensus regarding the most effective initiatives for reducing weight stigma within healthcare settings [26,82]. Connecting this to our findings, our study highlights a mother’s uncertainty in how to address the challenge of avoiding discussions about weight, as it is commonly assumed to be a significant aspect of health. Our research also shows that this assumption is firmly grounded in the children’s understanding of health. These examples highlight the complexities involved in implementing a more holistic healthcare approach that goes beyond traditional weight-centric discussions. 

### 5.4. The Necessity of Positivity and Relevance

Our results show that the relation between the families and healthcare professionals has a significant impact on the families’ perceptions of and attitudes toward health and weight management. Particularly, healthcare authenticity in the form of an overall approach seems highly appropriate to addressing and supporting the various emotions, challenges, and interactions that families encountered while participating in the program. This aspect was particularly reflected in the families’ experiences, as professionals experienced as being authentic contributed to effectively addressing and reducing negative feelings like guilt and shame. Moreover, experiencing authenticity motivated the families to actively engage in the program, leading them to reassess and modify their existing perceptions and approaches to health. This is in line with a study conducted by Hoeeg et al. [83], who explored how families perceived their involvement in a pediatric family-based childhood obesity intervention and identified a significant connection between the level of perceived authenticity and the level of adherence to the intervention.

However, it is also important to be aware of and recognize potential barriers that negatively influence the experience of pediatric health care, as these factors can contribute to the perception of stigma. As emphasized in the analysis, certain discussions about food and eating habits were observed to be topics that could trigger feelings of guilt and shame. The same applies to discussions about weight and the act of being weighed. These sensitive topics, closely related to feelings of guilt and shame, can quickly become stigmatizing practices, thereby contributing to the perception of stigma. Zenlea et al. [84] and Roberts et al. [56] have also revealed how healthcare professionals may unintentionally contribute to the perception of stigma by inadvertently reinforcing feelings of guilt and shame in their communication with children and families. 

In such situations, healthcare professionals must exercise extra care and vigilance in their interactions with families, challenging their familial self-perceptions and existing health expectations in a supportive manner.

## 6. Strengths and Limitations

The strengths of our study include the comprehensive theoretical framework combining theories on familial health identity and healthcare authenticity. Combining these theories provided a theoretically informed analytic framework that enabled us to examine the complexities of weight stigma in families with a child enrolled in a pediatric weight management program. This framework facilitated our exploration of how families negotiate and reconstruct health identities and how healthcare authenticity influences the perception and experience of weight stigma. By applying these theories, we demonstrate a robust theoretical and analytical framework that contributes to the currently sparse literature dedicated to exploring and identifying potential stigma-generating mechanisms.

Because we focused our research on joint narratives, it was important for us to interview the family members together. A potential drawback of this approach is that children might be influenced by their parents’ opinions and refrain from expressing their own. On the other hand, the emotional connections among family members are stronger than they are in a normal group interview. This heightened emotional bond is likely to elicit intimate narratives about their shared life experiences. In our case, the interviews proved particularly valuable for investigating the construction of narratives in real life, as they provided access to the ongoing development of these narratives in real time through interdependent relationships. Another limitation involves the potential for selection bias, wherein healthcare professionals involved in the program assisted in the recruitment of families. Nonetheless, this does not seem very likely, considering that the recruited families had both positive and negative feedback about the program.

We encouraged the participation of both parents, but only three fathers wanted to participate. This, in itself, constitutes a finding regarding who in the family is more involved in health and weight management, but it also means that our study lacks the fathers’ perspectives and experiences.

## 7. Implications for Practices

Our study highlights several key implications for pediatric healthcare practice in the context of addressing childhood obesity and managing weight-related issues within families. Overall, the four potential stigma-generating mechanisms we have identified in our study should constitute the main ingredients in stigma-preventive and/or stigma-reducing approaches to the treatment of childhood obesity: 

Promoting family-centered care*:* acknowledging the complexity of family responses to healthcare programs and the challenges families encounter when accepting assistance is crucial. Pediatric settings should provide resources and support to help families navigate these complexities, especially when their self-understanding is challenged. Furthermore, emphasizing family-centered care is highly essential. Addressing childhood obesity requires involving the entire family unit and providing support to both parents and children. By doing so, healthcare professionals can alleviate the individual burdens of guilt and shame that are often placed on parents and children.

Awareness of the effects of being weighed: our findings underscore the significance of understanding the potential implications of the weight concept. The families’ experiences reflect a favorable disposition toward shifting away from a solely weight-centric healthcare approach. Embracing a holistic health perspective that incorporates weight considerations without exclusively focusing on weight may aid in preventing and reducing the perception of stigma [85,86,87]. Additionally, involving families in conversations about how to communicate regarding weight and obesity is crucial, as these approaches may vary among different families.

Training and education with a particular focus on healthcare authenticity: healthcare professionals should prioritize being authentic when interacting with families—thereby creating a supportive and nonjudgmental environment. Authenticity can significantly influence how families perceive and engage with healthcare services. However, achieving this requires that healthcare professionals receive comprehensive training and education in understanding and addressing weight stigma. This training should encompass strategies for fostering authenticity, establishing a supportive environment, and engaging families in a non-stigmatizing manner. Additionally, pediatric healthcare settings must be mindful of potential barriers that can negatively affect the healthcare experience. Discussions related to sensitive topics such as food, eating habits, and weight can inadvertently reinforce feelings of guilt and shame. Therefore, care and sensitivity in these areas are crucial to prevent contributing to the perception of stigma.

## 8. Conclusions

Our study identified four key themes illustrating underlying mechanisms that can generate or amplify the perception of weight stigma within family and pediatric healthcare settings if not addressed promptly. It underscores the importance of adopting a holistic, family-centered, and authentic healthcare approach in addressing these nuanced challenges and reducing weight stigma.

## Figures and Tables

**Table 1:** Overview over the participants.

Families	Children	Mother	Father
**A**	Boy 12 years old	x	x
**B**	Girl 12 years old	x	x
**C**	Girl 9 years old	x	
**D**	Girl 11 years old	x	x
**E**	Boy 12 years old	x	
**F**	Boy 11 years old	x	
**G**	Girl 15 years old	x	
**H**	Boy 13 years old	x	
**I**	Boy 12 years old	x	
**J**	Girl 14 years old	x	
**K**	Girl 11 years old	x	

## Data Availability

The data presented in this study are available on request from the corresponding author. The data are not publicly available due to the need for anonymity.
